# 2378 Addressing challenges from missing data in a global quality improvement study

**DOI:** 10.1017/cts.2018.155

**Published:** 2018-11-21

**Authors:** Amelia Barwise, Lisha Yi, Jun Guo, Ognjen Gajic, Moldovan Sabov, Yue Dong, Rahul Kashyap

**Affiliations:** 1 Mayo Clinic; 2 Division of Pulmonary and Critical Care Medicine Mayo Clinic Rochester and Multidisciplinary Epidemiology and Translational Research in Intensive Care (METRIC) Group

## Abstract

OBJECTIVES/SPECIFIC AIMS: Missing data is a common problem in research studies that may lead to inconclusive or inaccurate results. It may even lead to harm secondary to wrong research conclusions. The purpose of this ancillary study is to measure the differences in missing data following implementation of a variety of mechanisms to improve data quality and documentation in a global quality improvement study. Many of the sites involved in the study were in low-income or middle-income countries with minimal research infrastructure. Missing data is defined as “values that are not available that would be meaningful for analysis if they were observed” (The prevention and treatment of missing data, *New Engl J Med* 367; 14, nejm.org, October 4, 2012). METHODS/STUDY POPULATION: All study sites used REDCap software to enter various data points including hospital and ICU admission and discharge dates as well as whether items on a Checklist relevant to processes of care in the ICU were reviewed. After initial general data collection phase, we categorized data as “must have” and “good to have.” “Must have” variables were defined as data variables that were essential for the study outcomes. “Good to have” variables would not affect the main outcomes of the study if missing. We measured completeness of data using the in-built REDCap data quality check feature. We used several strategies to encourage reduction of missing data. We initially did random data checks but noted that the amount of missing data was substantial and could not be adequately addressed this way. Second, we created excel sheets highlighting missing data for each site and notified sites. This proved onerous to create and made it burdensome for sites to identify easily where data was missing. Third, we built a custom report form in REDCap specifically able to identify which “must have” data points were missing. This could be easily accessed by the principal investigator at each site and made completing the data forms more straightforward. We encouraged all sites to complete their data collection by sending weekly data reports to each site highlighting the patients with missing data. An instructional YouTube tutorial was also created and the link was shared with all sites to demonstrate how to use the custom built report form in REDCap and how to appropriately fill in the missing data. Since this was a global study, we communicated with sites using a variety of locally favored mechanisms including Zoom, FaceTime, WeChat, WhatsApp as well as email. By harnessing the buy-in of local champions our approach was successful. RESULTS/ANTICIPATED RESULTS: The total number of patients recruited for the CERTAIN study is 4843. The rate of all missing variables improved with the efforts described above. Hospital admission dates were missing in 8.4% pre efforts and 4.2% post efforts (*p*<0.01). ICU admission dates were missing in 5.5% pre and 2.0% post (*p*<0.01). Documentation of completion of processes of care (including central line review, urinary catheter review, consideration for blood transfusion) improved significantly from pre to post (*p*<0.01). DISCUSSION/SIGNIFICANCE OF IMPACT: Missing data can be a problem in all types of research studies. This study provides some preliminary evidence for effective approaches that can reduce the problem of missing data when conducting a global study at sites with limited research infrastructure in place. By addressing the concern about missing data, we can be more confident that our results can be accurately analyzed and interpreted, improving the quality of the research.

